# Cis-regulatory sequences in plants: Their importance, discovery, and future challenges

**DOI:** 10.1093/plcell/koab281

**Published:** 2021-11-22

**Authors:** Robert J Schmitz, Erich Grotewold, Maike Stam

**Affiliations:** Department of Genetics, University of Georgia, Athens, Georgia 30602, USA; Department of Biochemistry and Molecular Biology, Michigan State University, East Lansing, Michigan 48824, USA; Swammerdam Institute for Life Sciences, University of Amsterdam, 1098 XH Amsterdam, The Netherlands

## Abstract

The identification and characterization of cis*-*regulatory DNA sequences and how they function to coordinate responses to developmental and environmental cues is of paramount importance to plant biology. Key to these regulatory processes are cis-regulatory modules (CRMs), which include enhancers and silencers. Despite the extraordinary advances in high-quality sequence assemblies and genome annotations, the identification and understanding of CRMs, and how they regulate gene expression, lag significantly behind. This is especially true for their distinguishing characteristics and activity states. Here, we review the current knowledge on CRMs and breakthrough technologies enabling identification, characterization, and validation of CRMs; we compare the genomic distributions of CRMs with respect to their target genes between different plant species, and discuss the role of transposable elements harboring CRMs in the evolution of gene expression. This is an exciting time to study cis-regulomes in plants; however, significant existing challenges need to be overcome to fully understand and appreciate the role of CRMs in plant biology and in crop improvement.

## Teaser

This review discusses the features of cis-regulatory sequences in plants, technologies enabling their identification, characterization, and validation, their organization into functional cis*-*regulatory modules, their genomic distributions with respect to target genes, and the role of transposable elements in their evolution.

## Introduction

**Figure 1:**
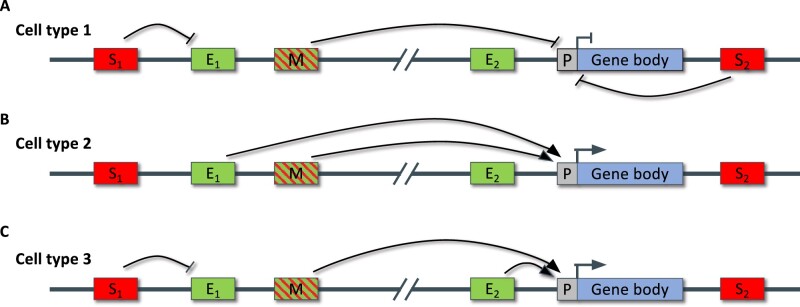
Combinatorial CRM actions elicit diverse transcriptional responses in distinct cell types. The activity of each CRM depends to a large extent on the expression levels of TFs that can bind the CRM. A, In cell type 1, silencer element 1 represses an enhancer, while a multifunctional sequence element and silencer element 2 repress promoter activity. B, In cell type 2, enhancer element 1 works cooperatively with a multifunctional sequence element to activate gene expression. C, In cell type 3, silencer element 1 represses the upstream enhancer 1, whereas the multifunctional sequence element activates the gene in concert with the promoter proximal enhancer (enhancer 2). S, silencer; E, enhancer; M, multifunctional sequence element; P, promoter.

A fundamental question in biology is how complex patterns of gene expression are regulated. Central to this is the genome-wide identification and characterization of cis-regulatory elements (CREs) and cis*-*regulatory modules (CRMs) that influence the expression of protein-coding and long noncoding RNA (*lncRNA*) genes (Shlyueva et al., 2014; Kopp and Mendell, 2018; Andersson and Sandelin, 2020; Della Rosa and Spivakov, 2020). We refer here to CREs as individual transcription factor (TF) binding sites, while CRMs are assemblies of CREs and include promoters, transcriptional enhancers, silencers, and insulator elements. CRMs determine in which cell, at what time, and at what level a gene is expressed (Table 1 and Figure 1). In animals, the generation of comprehensive chromatin and epigenome maps have made the identification of gene regulatory sequences routine, even though these DNA elements are often located kilobases to megabases away from their target genes (De Laat and Duboule, 2013; Shlyueva et al., 2014; Klemm et al., 2019). Epigenome maps are effective because regulatory sequences possess distinct chromatin signatures. For example, active regulatory sequences display accessible chromatin, TF binding, low DNA methylation, and histone modifications such as acetylation or methylation of specific lysine residues in histone H3 (Stadler et al., 2011; De Laat and Duboule, 2013; Shlyueva et al., 2014; Weber et al., 2016). Although relevant research in plants has lagged behind model animal species, recent studies in plants are revealing similar, but also distinct, molecular signatures at cis-regulatory sequences compared to animals. Although in plant species with smaller genomes, such as *Arabidopsis thaliana* (Arabidopsis), most CRMs are located in close linear proximity to the genes they control, the expansion of genome size and intergenic space in numerous plant species is associated with a large number of putative distal CRMs (Oka et al., 2017; Lu et al., 2019; Li et al., 2019a; Ricci et al., 2019). Distal CRMs can regulate genes tens or even hundreds of kilobases away, complicating their identification. The systematic discovery of CRMs and the CREs they are composed of is a first step in the engineering and rewiring of existing regulatory networks to optimize plant growth and development, enhance stress resilience or generate plant products. To maximize the use of natural variation, and for synthetic biology to realize its full potential, deeper knowledge of regulatory modules is paramount to facilitate crop improvement. Numerous reporter assays are now being developed and implemented for the identification and functional characterization of CRMs (Inoue and Ahituv, 2015; Ricci et al., 2019; Sun et al., 2019; Jores et al., 2020), but there is still a need for more epigenome maps from many different cell types and growth conditions.

**Table 1:** Major types of CRMs

Cis-regulatory element type	Definition
Core promoter	Minimal sequence region needed to direct initiation of transcription, usually spanning 50–100 bp around the TSS
Enhancer	A DNA sequence that, when bound by specific TFs and cofactors, increases the transcription initiation rate, and thereby the expression of target genes in a tissue-, developmental stage-, and/or condition-specific manner
Silencer	A DNA sequence that, when bound by specific TFs and cofactors, actively decreases the expression of target genes. A silencer might silence a gene directly, or indirectly by silencing enhancers
Insulator	An element located between CRMs and core promoters that, when bound by the appropriate proteins, prevents the activation or silencing of potential target genes by these CRMs. Such insulators are not known to exist in plants
Multifunctional sequence element	DNA element that exhibits more than one of the above properties at different times or conditions, or in different cells, e.g. enhancers in one cell type can function as silencers in other cell types and vice versa

## Transcription factor binding sites: important CRM constituents

CRMs are assemblies of CREs, which serve as sequence-specific binding sites for TFs and are key components of the regulatory portion of each eukaryotic genome. The combination of TFs expressed and bound to CRMs in a particular cell type determines the activity and the ultimate function of CRMs (Kaufmann et al., 2010; Lambert et al., 2018). To activate CRMs, pioneer TFs (described below) bind to nucleosomal DNA, and recruit histone-modifying enzymes, such as histone acetyltransferases, and chromatin remodeling complexes that open the chromatin structure by displacing linker histones and mediating nucleosome eviction (Cirillo et al., 2002; Spitz and Furlong, 2012; Pajoro et al., 2014; Iwafuchi-Doi et al., 2016; Jin et al., 2021). Subsequently, other nonpioneer TFs and co-factors can bind to DNA within the accessible chromatin regions (ACRs), ultimately resulting in fully activated CRMs, which physically interact with their target genes through protein–protein interactions to regulate their transcription levels (Nolis et al., 2009; Quevedo et al., 2019).

**Figure 2:**
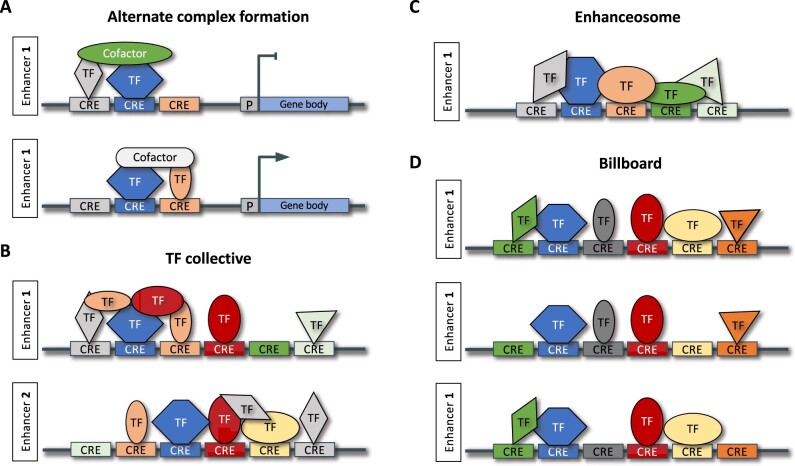
Models for enhancer organization. A, Example of alternative complex formation. TFs can function both as transcriptional activators and repressors, depending on the proteins (co-activators or co-repressors) they interact with. B, Examples of TF collective enhancers. This model is characterized by cooperative DNA-binding, which can be achieved by many different mechanisms, and by proteins as well as DNA serving as a scaffold for the binding of TFs. It allows for flexible CRE arrangements, resulting in distinct regulatory outputs (exemplified by the two enhancers shown). C, Example of an enhanceosome enhancer. In the enhanceosome model, the binding of the various TFs to the respective CREs must occur in a specific order and orientation, following a particular grammar. D, Example of billboard enhancers. In the billboard model, the composition as well as the position and orientation of CREs within an enhancer is preserved. The regulatory output differs depending on the expression and activity level of TFs that can bind the CREs. Key: The thick gray line represents DNA, with the colored rectangles indicating CREs. Each different color indicates a different DNA motif. TFs are depicted in various shapes and colors, with each color denoting a different TF that recognizes a specific DNA sequence (matching colors).

In eukaryotes, 5%–7% of all nuclear genes encode TFs, proteins that recognize short DNA motifs in a sequence-specific fashion (Riechmann and Ratcliffe, 2000; Lambert et al., 2018). Most TF-binding sites (TFBSs) are small (6–12 bases) and are therefore represented thousands of times in any eukaryotic genome (Wunderlich and Mirny, 2009; Lambert et al., 2018). TFs are usually characterized by the presence of one or more conserved DNA-binding and/or dimerization domains that allow grouping them into 40 or more families (Yilmaz et al., 2009; Riechmann and Ratcliffe, 2000; Lambert et al., 2018). Traditionally, TFs have been classified into activators or repressors, but it is clear that many can function as both activator and repressor, depending on the proteins (co-activators or co-repressors) they interact with (alternate complex formation; Figure 2A;  Rosenfeld et al., 2006; Ikeda et al., 2009; Lambert et al., 2018; Haberle and Stark, 2018). For example, Arabidopsis WUSCHEL, a central regulator of stem cell proliferation, functions primarily as a repressor, yet has the ability to activate transcription of the floral homeotic gene *AGAMOUS (AG*; Ikeda et al., 2009).

Identifying the sequences regulated by specific TFs is an area of active research. Members from the same TF family often recognize very similar DNA sequences in vitro (Weirauch et al., 2014), but with different DNA-binding affinities. Differences in DNA-binding preferences between paralogous TFs are important in establishing in vivo binding selectivity (Shen et al., 2018; Panchy et al., 2019). Predicting which specific TF is tethered to a particular CRE in the genome in a given cell type remains challenging. Predictions require a good understanding of the cell types in which a TF is expressed and at what levels compared with the putative target genes; whether it is a pioneer or nonpioneer TF, and whether or not corresponding CREs are accessible. Consequently, it remains difficult to assign a role in activating or repressing transcription to a particular CRE in the genome. This difficulty is further augmented by multiple CREs together forming a CRM.

For most TFs, nucleosomes provide a challenging barrier to access the CREs they preferably bind to (Bai and Morozov, 2010; Zaret and Carroll, 2011; Zaret and Mango, 2016). In contrast to most TFs, pioneer TFs have the ability to bind their cognate TFBSs on nucleosomes in inaccessible chromatin, enabling CRMs to adopt a state of competence to activate or repress transcription by recruiting activators or repressors, respectively (Zaret and Carroll, 2011; Pajoro et al., 2014; Zaret and Mango, 2016; Tao et al., 2017; Jin et al., 2021). The first identified pioneer TF, FoxA (*forkhead box A*), involved in the development of endoderm-derived organs during metazoan embryo development (Lee et al., 2005), takes advantage of the similarity of its “winged-helix” DNA binding domain to linker histone H1 to displace H1 and gain access to nucleosomal DNA (Clark et al., 1993; Iwafuchi-Doi et al., 2016). Note that pioneer TFs use a variety of mechanisms to bind to DNA (Zaret and Carroll, 2011; Zaret and Mango, 2016). Even DNA methylation, generally associated with inaccessible chromatin, can provide an anchor point for pioneer TFs. Indeed, many pioneer TFs, including FoxA, bind methylated DNA (Zhu et al., 2016). In plants, several pioneer TFs have been identified, including LEAFY COTYLEDON1, LEAFY, APETALA1, and SEPALLATA3, which are respectively involved in controlling Arabidopsis flowering competency, flower meristem establishment, and floral organ specification (Pajoro et al., 2014; Tao et al., 2017; Jin et al., 2021).

## CRMs: characteristics of the main players

CRMs and their activity states are characterized by different combinations of DNA and chromatin features, whereby the features of promoters and enhancers are more well-defined than those of silencers (Pajoro et al., 2014; Shlyueva et al., 2014; Weber et al., 2016; Marand et al., 2017; Xiao et al., 2017; Oka et al., 2017; Ricci et al., 2019; Andersson and Sandelin, 2020; Gisselbrecht et al., 2020; Pang and Snyder, 2020; Zhao et al., 2020). In plants, the DNA of the vast majority of CRMs appears stably unmethylated in a tissue-independent manner, and these unmethylated regions (UMRs) are enriched in accessible chromatin, histone acetylation (HAc), and TF–DNA interactions (Schmitz et al., 2013; Li et al., 2015; Kawakatsu et al., 2016; Oka et al., 2017; Ricci et al., 2019; Crisp et al., 2020). Much of the rest of the genome is methylated, including a subset of genes and transcriptionally silenced transposable elements (TEs; Zhang et al., 2006; Zilberman et al., 2007; Gent et al., 2013; Niederhuth et al., 2016). Therefore, UMRs likely encompass the vast majority of the CRMs within plant genomes, independent of their activity. This is consistent with the majority of Arabidopsis TFs not binding to methylated cytosines (O’Malley et al., 2016). A small fraction of UMRs appear tissue-specific, however (Oka et al., 2020). Indeed, active DNA demethylation at CRMs has been shown to play a role in specific developmental processes, such as fruit ripening in tomato and plant responses to stress conditions (Yua et al., 2013; Zhong et al., 2013; Liu et al., 2015; Deleris et al., 2016; Halter et al., 2021).

**Figure 3:**
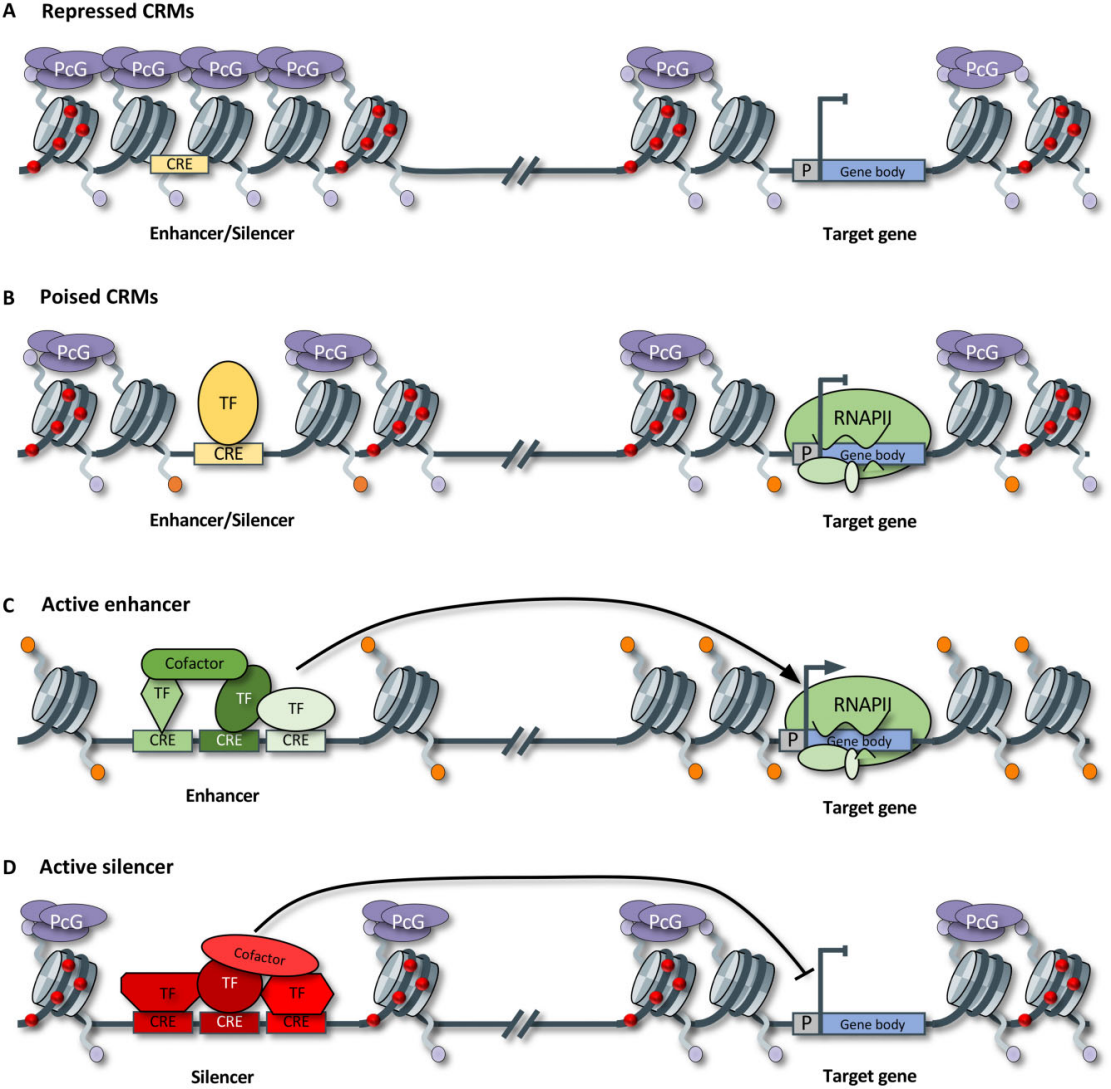
Chromatin accessibility and modifications associated with different CRM activities. A, Example of a repressed CRM. CREs are occluded by nucleosomes due to inaccessible chromatin imparted by PcG protein complexes and H3K27me3. This is associated with transcriptional silencing of the target gene. B, Example of a poised CRM, where nucleosomes flanking CREs have low histone acetylation levels and only a few CREs are accessible for TF binding. PcG proteins and H3K27me3 are still present. The promoter is engaged by RNAPII, and flanking nucleosomes have low histone acetylation levels. PcG proteins and H3K27me3 hamper RNAPII elongation and transcript production. C, Example of an active enhancer, whereby multiple TFs and cofactors interact with a CRM, and target the promoter to activate transcription of the target gene. Nucleosomes are enriched for histone acetylation. D, Example of an active silencer where TFs and cofactors bind to the CRM to recruit PcG proteins to catalyze H3K27me3 and silencing of the target gene. Purple ovals, PCG protein complexes; P, promoter; RNAPII, RNA polymerase II; Purple circles, H3K27me3; orange circles, histone acetylation; red circles, DNA methylation.

Several different activity states have been described for CRMs, but as not all of these are defined unequivocally, we limit our description to the repressed, poised, and active states. Active CRMs are accessible for DNA-protein interactions, and enriched with (among others) HAc, which weakens the electrostatic interactions between nucleosomal DNA and histones, improving accessibility for the transcription machinery (Zhang et al., 2015; Fenley et al., 2018). Based on current knowledge, chromatin of repressed CRMs is inaccessible, unbound by TFs, and enriched with trimethylation of histone H3 lysine K27 (H3K27me3; Shlyueva et al., 2014; Zhang et al., 2015). H3K27me3 marks transcriptionally silenced genes, their proximal flanking regions, and distal intergenic regions, and is associated with the presence of Polycomb Group (PcG) protein complexes (Simon and Kingston, 2013; Wang and Shen, 2018; Zhao et al., 2018; Huang et al., 2019; Lu et al., 2019; Ricci et al., 2019; Ngan et al., 2020; Pang and Snyder, 2020). Poised CRMs have an activity state between repressed and active and are ready to become fully activated or inactivated (Rada-Iglesias et al., 2011; Koenecke et al., 2017). They are accessible, bound by few TFs, and enriched with H3K27me3 and low levels of an active histone modification, such as HAc (Figure 3;  Ernst et al., 2011; Rada-Iglesias et al., 2011; Huang et al., 2019; Lu et al., 2019; Ricci et al., 2019). In animals, poised and active CRMs are generally associated with H3K4me1 (Rada-Iglesias et al., 2011), but this does not seem the case for plants (Oka et al., 2017; Li et al., 2019b; Ricci et al., 2019; Sun et al., 2019).

In various organisms, active enhancers and silencers produce transcripts, including enhancer RNAs and lncRNAs; de Santa et al., 2010; Kim et al., 2010; Quinn and Chang, 2016; Oka et al., 2017; Ricci et al., 2019; Gil and Ulitsky, 2020; Pang and Snyder, 2020). Knowledge of CRM-derived RNAs in plants is limited. In animals, enhancer RNAs are usually unstable, and produced in both directions (bidirectional) at the border of ACRs, at significantly lower levels than the unidirectional transcripts directed by core promoters (Andersson et al., 2014a; Core et al., 2014; Andersson and Sandelin, 2020). For mammalian cells, enhancer RNAs are used to predict enhancers (Andersson et al., 2014a), and a number of enhancer-derived lncRNAs play a role in enhancer function (Quinn and Chang, 2016; Gil and Ulitsky, 2020). However, the level at which transcription occurs at enhancers in plants is unclear. In Arabidopsis, transcription at intergenic regions seems rare (Hetzel et al., 2016; Thieffry et al., 2020). In maize, several dynamically expressed high-confidence lncRNAs associated with ACRs have been described, and co-expressed lncRNA–protein-coding gene pairs identified, suggesting a regulatory relationship; a small fraction of the lncRNAs overlapped with putative enhancers discovered in maize (Oka et al., 2017; Parvathaneni et al., 2020). It is important to note that for less well-annotated genomes, lncRNAs may correspond to unannotated protein-coding genes (Monnahan et al., 2020; Mendieta et al., 2021).

Most characteristics are not limited to particular types of CRMs, hampering identification through the use of single characteristics. Instead, combinations of descriptors are often used to distinguish different types of CRMs. For example, in animals, high and low H3K4me3/H3K4me1 ratios are indicative of core promoters and enhancers, respectively.

### Promoters

For historical reasons, the term promoter is often used to indicate one to two kilobase pairs (kbp) upstream of the transcription start site (TSS; Juven-Gershon and Kadonaga, 2010). Such regions may contain enhancer and/or silencer elements, obscuring the discussion of the different types of regulatory elements. Therefore, in this review, we define a promoter as what is also known as the core promoter, which is the minimal sequence region that is needed to direct initiation of transcription, usually spanning 50–100 base pairs (bp) around the TSS. For RNA polymerase II (RNAPII) transcribed genes, this is the region to which the general TFs bind to help facilitate RNAPII to initiate transcription (Haberle and Stark, 2018; Andersson and Sandelin, 2020). General TFs are defined by their binding to core promoters to help position RNAPII. The core promoter alone drives only low (basal) transcription levels, and sometimes contains a TATA box (Das and Bansal, 2019). The TATA box is recognized by the TATA-box binding protein, a key factor in recognizing core promoter elements (Haberle and Stark, 2018; Ngoc et al., 2019). In plants, however, the majority of the core promoters are TATA-less; at such promoters other cis-elements play a role in transcription initiation (Molina and Grotewold, 2005; Lenhard et al., 2012; Mejía-Guerra et al., 2015). Besides a TATA box, cis-elements such as a CCAAT-box and TC-elements are shown to contribute to the correct positioning of the transcription machinery and efficient transcription initiation (Dolfini et al., 2009; Bernard et al., 2010; Laloum et al., 2013; Porto et al., 2014).

Active animal and plant promoters are characterized by accessible chromatin, H3K4me3, HAc, H2A.Z, a lack of DNA methylation, the binding of general TFs and RNAPII, and the production of stable, unidirectional transcripts (Heintzman et al., 2007; Zilberman et al., 2007; Stadler et al., 2011; Zhang et al., 2012b, 2006; Ricci et al., 2019; Andersson and Sandelin, 2020; Oka et al., 2020; Thieffry et al., 2020). Generally, promoters display a positive correlation between transcript levels and the levels of H3K4me3 and HAc and are associated with reduced nucleosomal occupancy (Barski et al., 2007; Zhang et al., 2012b; Core et al., 2014; Oka et al., 2017; Yan et al., 2019). Inactive promoters are generally enriched for H3K27me3 and are less accessible (Ernst et al., 2011; Lu et al., 2019; Ricci et al., 2019). Little is known about poised plant promoters. They may be marked by HAc, but also RNAPII (Offermann et al., 2008; Gagete et al., 2011), whereas the combination of H3K27me3 with H3K4me3 found in animals may be lacking (Mikkelsen et al., 2007; Zeng et al., 2019; Blanco et al., 2020). Typically, to allow transcription initiation, promoter DNA must be unmethylated, irrespective of its activity (Appanah et al., 2007; Zilberman et al., 2007; Zemach et al., 2010; Stadler et al., 2011).

### Enhancers

Transcriptional enhancers are CRMs that, when bound by specific TFs and cofactors, increase the transcription initiation rate, and thereby the expression of their target genes in a tissue-, developmental stage-, and/or condition-specific manner. Enhancers can be located more than 1 Mbp away from their target gene (Lettice et al., 2002; Benko et al., 2009); the average distance depends on the size of the genome (Lu et al., 2019). The first distal enhancer discovered in plants was the maize *b1* (*booster1*) hepta-repeat enhancer, located ∼100-kbp upstream of the TSS of the *b1* gene (Stam et al., 2002; Belele et al., 2013). The most distal enhancer so far described in plants is DIstal Cis-Element (DICE), which is required for high expression of the maize *bx1 (benzoxazinless1)* gene ∼140 kbp away (Zheng et al., 2015). Enhancers have been defined to increase reporter gene expression when located up- or downstream of the gene, in an orientation independent manner (Shlyueva et al., 2014; Andersson and Sandelin, 2020). Recent data indicate the latter may, however, not be true for all enhancers (Mikhaylichenko et al., 2018; Ricci et al., 2019; Jores et al., 2020). Active enhancers usually regulate their target genes through chromatin interactions, which involve TFs and cofactors at the enhancer, and the transcription initiation complex at the promoter (De Laat and Duboule, 2013; Long et al., 2016; Peng et al., 2019; Li et al., 2019a; Doni Jayavelu et al., 2020; Ngan et al., 2020). LncRNAs have also been implicated in these interactions (Li and Fu, 2019).

The billboard, enhanceosome, and TF collective models represent three different mechanisms for the interplay between enhancers and TFs, resulting in active regulatory sequences (Figure 2, B–D). In the billboard model, neighboring CREs form a CRM, but the individual CREs can almost independently affect gene expression; TFs can bind cooperatively and additively to the individual CREs and the composition of bound TFs is interpreted by the basal transcription machinery (Kulkarni and Arnosti, 2003; Spitz and Furlong, 2012). This enables the same CRM to have different effects in different cell types depending on the expressed and bound TFs. In the enhanceosome model, enhancers are organized using a particular grammar of TF binding motifs; a specific order and orientation of CREs is necessary for the cooperative binding of specific TFs, and thereby for enhancer function (Spitz and Furlong, 2012; Weingarten-Gabbay and Segal, 2014; Vockley et al., 2017). In the TF collective model, the recruitment of TFs is accomplished through binding to CREs and protein–protein interactions (Junion et al., 2012; Long et al., 2016; Figure 2). While specific details of the different models vary in the literature (Spitz and Furlong, 2012; Long et al., 2016), the picture is emerging that TFs are tethered to enhancers using additive or cooperative, and direct or indirect DNA binding (Heyndrickx et al., 2014). Note that while these models have been proposed for enhancers, they are likely to be valid for silencers as well.

Genes can be regulated by multiple enhancers that act either complementarily, redundantly, pleiotropically, interdependently, or synergistically (Figure 1;  Cannavò et al., 2016; Osterwalder et al., 2018; Lewis et al., 2019; Sabarís et al., 2019). For example, different enhancers at the maize *b1* gene display a different tissue-specificity (Stam et al., 2002), and the Arabidopsis *SHATTERPROOF2* gene is regulated by two redundant CRMs (Bhupinder and Franks, 2017). In *Solanum lycopersicum*, deleting combinations of candidate CRMs revealed both additive and synergistic interactions in the production of locule number depending on which CRM was deleted (Wang et al., 2021). Comparable to how genes can be targeted by multiple enhancers, enhancers can interact with more than one target gene, and are able to “skip over” genes, interacting with genes further away, in terms of the linear sequence, than the nearest flanking gene (Ghavi-Helm et al., 2014; Peng et al., 2019; Li et al., 2019a; Ricci et al., 2019). For example, in maize, DICE enhances expression of *bx1*, but not *bx8*, which is located between the two (Zheng et al., 2015).

Similar to promoters, enhancers can be in active, repressed, or poised states (Figure 3). Active enhancers typically are characterized by the binding of activating TFs and cofactors, a lack of DNA methylation, ACRs with HAc (e.g. H3K9ac, H3K27ac, H3K56ac), H2A.Z at flanking nucleosomes, and physical interactions with target genes (Zhang et al., 2012b; Zhu et al., 2015; Oka et al., 2017; Li et al., 2019a; Peng et al., 2019; Ricci et al., 2019; Crisp et al., 2020; Zhao et al., 2020a). Enhancers are often tissue-specific; in the tissues they are not active, they are likely repressed or poised. We hypothesize that most repressed enhancers are inaccessible and enriched for repressing modifications, such as H3K27me3, and need to be bound by pioneer TFs to become accessible and ready for activation (Figure 3;  Pajoro et al., 2014; Zhu et al., 2015; Sun et al., 2019). Poised enhancers may be bound by a few TFs, have ACRs, and be enriched for both H3K27me3 and low HAc levels (Koenecke et al., 2017; Ricci et al., 2019; Sun et al., 2019). Upon activation, H3K27me3 is removed and activating histone modifications added.

There may be other enhancer activity states besides those discussed above. Indeed, the largest class of distal accessible, UMRs identified in various plants is characterized by the absence of the histone modifications tested for (Lu et al., 2019; Ricci et al., 2019; Oka et al., 2020), yet the histone variant H2A.Z is still present at these regions (Lu et al., 2019; Ricci et al., 2019). In maize, such “unmodified regions” display enhancer activity in transient assays, albeit at lower average levels than ACRs marked with HAc (Ricci et al., 2019). Gene ontology enrichment analysis of their flanking genes suggests these regions may be involved in developmental programs. In addition, there may be DNA-methylated CRMs within TEs that are demethylated in specific cell types or upon stress treatment, affecting the expression of genes involved in among others plant disease resistance (Deleris et al., 2016; Halter et al., 2021).

### Silencers

Silencers, when bound by TFs and associated cofactors, actively decrease the expression of their target genes (Figures 1 and 3). They are crucial for establishing precise, tissue-specific expression patterns by blocking expression in cell types and tissues where the gene should be silenced; they prevent ectopic gene expression (Ogbourne and Antalis, 1998). For example, a 100-bp fragment in the second intron of the tobacco *AG* gene represses *AG* expression in nonfloral tissues, promoting flower-specific *AG* expression (Liu et al., 2020). Similarly, TACPyAT repeats in the *Petunia hybrida chalcone synthase A* gene promoter hamper ectopic *chalcone synthase A* expression (van der Meer et al., 1992). Like enhancers, silencers are also proposed to act in a position- and orientation-independent manner (Figure 1) (Laimins et al., 1986; Sawada et al., 1994; Ogbourne and Antalis, 1998). Silencers can have multiple action modes. They can silence a gene directly, but also indirectly by silencing enhancers (Figure 1; Harris et al., 2016; Ngan et al., 2020; Pang and Snyder, 2020).

Although silencers were described decades ago (Laimins et al., 1986; Kuhlemeier et al., 1987; van der Meer et al., 1992; Sawada et al., 1994; Liu et al., 2011), attention to silencers has only increased recently (Oki and Kamakaka, 2002; Chetverina et al., 2014; Xiao et al., 2017; Huang et al., 2019; Doni Jayavelu et al., 2020; Gisselbrecht et al., 2020; Ngan et al., 2020; Pang and Snyder, 2020). These recent studies indicate that silencers, like enhancers, can inactivate genes from distal genomic locations, and can regulate multiple genes. Furthermore, a single gene can be controlled by multiple silencers. Future experiments will be needed to determine if this is also true for plants. Intriguingly, silencers can exhibit enhancer activity in other cell types, indicating multifunctionality (see below).

Silencers, like enhancers, probably exist in multiple activity states (Figure 3). The common denominator for active silencers across various genome-wide studies seems to be accessible chromatin enriched for H3K27me3. Xiao et al. (2017) indeed showed that Arabidopsis Polycomb response elements recruit Polycomb proteins and H3K27me3, and silence nearby genes in a Polycomb-dependent manner (Xiao et al., 2017). Also, several maize ACRs enriched with H3K27me3 were associated with transcriptional repression of the closest genes (Ricci et al., 2019); although it remains to be investigated if these ACRs act as silencers. However, depending on the pre-established bias towards features silencers are expected to display, the chromatin characteristics of identified silencers differ, suggesting the existence of different classes of silencers. In a study focusing on human and mice, the vast majority of ACR-based silencers were not enriched with H3K27me3; some were enriched for H3K9me3 (Doni Jayavelu et al., 2020). In *Drosophila melanogaster* none of 20 preselected H3K27me3-enriched ACRs showed silencing activity (Gisselbrecht et al., 2020). Different combinations of histone marks have been observed at silencers, H4K20me with H3K27me3 or H3K9me3, or H3K27me3 with active marks such as H3K4me1, H3K27ac, H3K4me3, or H3K9ac (Ngan et al., 2020; Huang et al., 2019; Pang and Snyder, 2020; Gisselbrecht et al., 2020). It was therefore concluded that to date there is no combination of chromatin marks that can unequivocally discriminate between silencers and other CRMs (Gisselbrecht et al., 2020). The identification and characterization of TFs binding to individual CREs within CRMs is likely key to distinguish different types of CRMs (Doni Jayavelu et al., 2020; Gisselbrecht et al., 2020). For example, in Arabidopsis, knockdown of two TFs binding to selected PREs yielded significantly higher expression levels of the target genes tested (Xiao et al., 2017).

### Insulators

Insulators or boundary elements are another type of CRM studied extensively in animals (Oki and Kamakaka, 2002; Chetverina et al., 2014). An insulator is defined as a DNA sequence that, when bound by the appropriate proteins and located between CRMs and core promoters, prevents the activation or silencing of potential target genes by these CRMs. They also prevent the inactivation of active genes by nearby heterochromatin. The most studied insulator protein is the CCCTC-binding factor (CTCF) which, however, is absent in plants (Heger et al., 2012). Accordingly, there are currently no indications that genuine insulator elements exist in plants (Heger and Wiehe, 2014). Plant genomes are able to maintain proper gene expression patterns without classic insulator sequences or proteins. It has been indicated that RNA-directed DNA methylation of sequences between euchromatin and heterochromatin provides local boundary activity (Li et al., 2015). Note that “insulator” is a term used otherwise by synthetic biologists; in their transgene design it refers to “transcription blockers” and/or “transcription terminators” (Schaumberg et al., 2015).

### Multifunctional sequence elements

In reality, the different functionalities of CRMs as described above are often intermingled rather than clearly separated (Figure 1). In animals, it has been shown that some core promoters can display enhancer activity and even regulate the expression of genes other than those for which it acts as core promoter (Dao et al., 2017; Diao et al., 2017; Mikhaylichenko et al., 2018; Andersson and Sandelin, 2020). Diao et al. (2017), for example, observed that core promoters of multiple genes enhanced expression of the *Pou Class 5 Homeobox 1* gene in human embryonic stem cells. Recent results also suggest enhancer activity by core promoters in plants (Sun et al., 2019). However, this study tested randomly sheared ∼670-bp genomic regions. Therefore, re-evaluation using smaller sequence regions restricted to just core promoters is needed.

Conversely, enhancers often mediate the production of transcripts by RNAPII, indicating promoter activity (Andersson et al., 2014b; Mikhaylichenko et al., 2018; Andersson and Sandelin, 2020). Indeed, enhancers can carry sequence elements resembling those of core promoters, including TATA-box-like motifs (Andersson et al., 2014a; Core et al., 2014). Transcription of intergenic regions has also been observed in plants (Oka et al., 2017; Ricci et al., 2019; Thieffry et al., 2020). However, it is unclear how many of these intergenic regions are mis-annotated genes.

DNA elements acting as enhancers in one cell type can function as silencers in other cell types and vice versa (Doni Jayavelu et al., 2020; Gisselbrecht et al., 2020; Ngan et al., 2020; Pang and Snyder, 2020). This intermingling of enhancer and silencer functions in one and the same CRM is likely due to combinations of binding sites for TFs mediating either of the activities. In such cases, the function shown in a particular cell type will depend on the expression levels of the corresponding TFs. An intricate interplay between intermingled enhancer and silencer functions is suggested to fine-tune target gene expression and define the boundaries between cells that do or do not express a given target gene (Huang et al., 2019; Lewis et al., 2019). Lewis et al. (2019) proposed that complex expression patterns evolved through combining enhancers mediating broad expression patterns with repressor binding sites that silence enhancer activity in specific cells.

## Identifying and verifying CRMs individually and genome-wide

Pinpointing and characterizing CRMs and their target genes is a challenging endeavor; however, there are a variety of approaches that have proven useful. The two main approaches, targeted and genome-wide, are briefly discussed below.

### Targeted identification of CRMs

Once transgenesis of chimeric sequences was established in plants, discovery of tissue-specific and environmentally responsive CRMs followed (Timko et al., 1985; van der Meer et al., 1992). One approach to identify unknown CRMs was the development of transgenic enhancer-trap lines (Sundaresan et al., 1995; Wu et al., 2003; Gardner et al., 2009). Several lines with tissue-specific expression patterns were identified. One example of a successfully identified enhancer is the Arabidopsis *MATURE MINOR VEIN ELEMENT1* (McGarry and Ayre, 2008).

Although the above-mentioned techniques are useful when dealing with easily transformable, small-genome organisms, for large-genome organisms, such as maize, other methods appear more useful. Many of the best examples were discovered using quantitative trait loci (QTL) mapping and genetic fine mapping approaches, for example, in maize, the tandem repeat *b1* enhancer, the *teosinte branched 1 (tb1)* enhancer, and *Vegetative to generative transition 1 (Vgt1)*, and DICE, mapping ∼100, ∼70, ∼60, and ∼140-kbp upstream of their (presumed) target genes, respectively (Stam et al., 2002; Clark et al., 2006; Salvi et al., 2007; Studer et al., 2011; Zheng et al., 2015). Interestingly, *KERNEL ROW NUMBER4* (*KRN4)*, a recently identified enhancer, is located ∼60-kbp downstream of its target gene, *UNBRANCHED3* (*UB3*) (Du et al., 2020). The *b1*, *tb1*, and *KRN4* enhancers were validated in transgenic and transient reporter assays (Studer et al., 2011; Belele et al., 2013; Du et al., 2020). These *tour de force* efforts to identify the causal basis for QTL are excellent examples of how distal CRMs are important to natural phenotypic variation in maize.

### Genome-wide identification and characterization of CRMs

The advent of microarray and high-throughput sequencing boosted approaches to identify CRMs genome-wide tremendously (Heintzman et al., 2007; Visel et al., 2009; Rada-Iglesias et al., 2011). In particular, epigenomic features, TF binding, and chromatin interactions have proven useful for CRM detection (Lieberman-Aiden et al., 2009; Bernstein et al., 2010; Spitz and Furlong, 2012; Shlyueva et al., 2014; Weber et al., 2016; Oka et al., 2017; Lu et al., 2019; Ricci et al., 2019). Of note is that strategic combinations of features are needed to identify and characterize CRMs and their target genes (Oka et al., 2017; Ricci et al., 2019; Chandra et al., 2021; Gisselbrecht et al., 2020).

The absence of DNA methylation can be used to predict plant CRMs in a genome-wide, tissue-independent manner (Oka et al., 2017, 2020; Ricci et al., 2019; Crisp et al., 2020), particularly when dealing with large, highly methylated genomes, while tissue/single-cell assays will pinpoint when and in which plant tissues/cell-types these CRMs may be active (Marand et al., 2021). Single-base resolution DNA methylation data are generated by whole-genome bisulfite sequencing (Cokus et al., 2008; Lister et al., 2008), or through the recently developed enzymatic methyl sequencing (Feng et al., 2020).

For the tissue-specific, genome-wide identification of ACRs, numerous assays exist, including DNase I-sequencing (Boyle et al., 2008), formaldehyde-assisted isolation of regulatory element-sequencing (Giresi et al., 2007), micrococcal nuclease-sequencing (Schones et al., 2008), and assay for transposase accessible chromatin-sequencing (ATAC-seq; Buenrostro et al., 2013). The advantage of ATAC-seq is that it requires relatively low number of cells compared to other assays (Lu et al., 2017). Importantly, single-cell ATAC-seq in plants proved highly effective for the detection of CRMs at single-cell resolution (Marand et al., 2021), allowing the detection of cell-type-specific CRMs, even in lowly abundant cell types. Data from Arabidopsis and maize revealed that approximately one-third of detected ACRs are cell-type specific (Dorrity et al., 2021; Marand et al., 2021). Notably, the latter were enriched for TFBSs of TFs expressed in these cells (Dorrity et al., 2021; Marand et al., 2021).

Chromatin immunoprecipitation-sequencing (ChIP-seq; Haring et al., 2007; Johnson et al., 2007) is a widely used technique to detect and characterize CRMs. It identifies features of interest such as histone modifications, histone variants, TFs, transcriptional cofactors, and RNA polymerase (Shlyueva et al., 2014). Histone modifications in particular are instrumental in distinguishing between activity states of plant CRMs.

Putative intergenic CRMs are often evolutionarily constrained (Studer et al., 2011; Rodgers Melnick et al., 2016; Lu et al., 2019; Ricci et al., 2019). Thus, identification of conserved noncoding sequences (CNS) by comparative genomic approaches can be used to identify CRMs (Van de Velde et al., 2016). A major challenge in identifying CNS is the generally short length of the CREs within CRMs, in combination with the higher sequence turnover of sequences flanking CREs (Van de Velde et al., 2016).

To identify putative CRMs underlying expression or phenotypic variation, existing genetic variation can be used (Rodgers Melnick et al., 2016; Ricci et al., 2019; Parvathaneni et al., 2020; Zhou et al., 2021). For example, genome-wide association studies and expression QTL analyses have been used to detect genotype-trait and expression associations, respectively, in noncoding regions in plant species (Zhang et al., 2011; Josephs et al., 2015; Rodgers Melnick et al., 2016; Kremling et al., 2018). In maize, ∼40% of the heritable phenotypic variance underlying certain complex traits is found in ACRs and likely due to variation in CRMs (Rodgers Melnick et al., 2016). To evaluate the possible functional consequences of nucleotide polymorphisms within putative CRMs, the integration with multiple data types, including epigenomic data, can be used (Joly-Lopez et al., 2020).

Numerous CRMs and their target genes are located more than 10 kbp apart (Wang et al., 2017; Oka et al., 2017; Li et al., 2019b; Lu et al., 2019; Ricci et al., 2019), complicating the identification of CRM–gene pairs. In this respect, advantage may be taken of the physical chromatin interactions occurring between CRMs and their target genes. Such interactions are usually identified using proximity ligation techniques commonly known as chromosome conformation capture (3C)-based techniques (Louwers et al., 2009; de Wit and de Laat, 2012; Hughes et al., 2014; Mumbach et al., 2016; Wang et al., 2017; Lin et al., 2019, 2018; Peng et al., 2019; Ricci et al., 2019; Li et al., 2019a; Gisselbrecht et al., 2020; Sun et al., 2020). Hi-C can reveal genome-wide chromatin interactions, especially in plants with relatively small genomes. In plants with large genomes, alternative methods such as chromatin interaction analysis by paired-end tag (ChIA-PET) and Hi-ChIP were recently adopted to enrich specific subsets of interactions associated with particular histone modifications or proteins present at CRMs (Li et al., 2019a; Peng et al., 2019; Ricci et al., 2019; Zhao et al., 2019a). For example, ChIA-PET and Hi-ChIP using antibodies against RNAPII indicated promoter–promoter (P–P) interactions in rice, wheat, and maize (Peng et al., 2019; Li et al., 2019a; Zhao et al., 2019a; Concia et al., 2020), while antibodies against H3K27ac and H3K27me3 may indicate active enhancers, and inactive enhancers or silencers, respectively (Li et al., 2019a; Ricci et al., 2019).

### Validation of CRM functions

**Figure 4:**
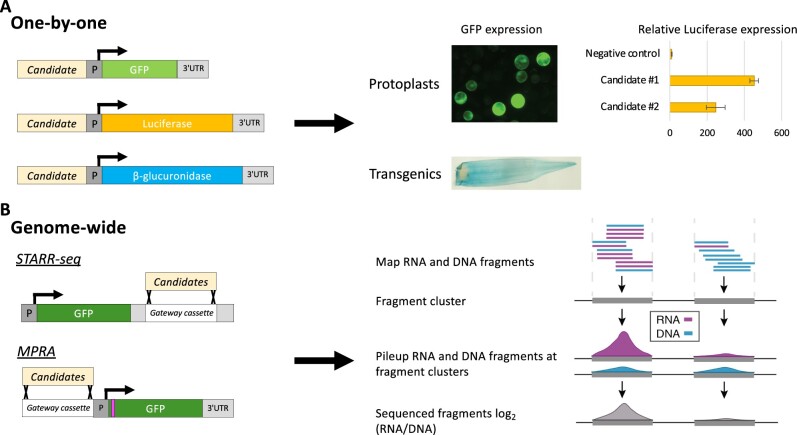
Evaluation of CRM activity using reporter assays. A, Candidate cis-regulatory sequences (candidate) can be tested for enhancer activity by fusion with a minimal promoter and reporter gene such as GFP, β-glucuronidase, or luciferase (left). Constructs are either transiently transfected into protoplasts or stably integrated into a plant genome to evaluate reporter gene activity (right). Some assays, for example luciferase assays, provide quantitative read-outs. Activities need to be examined relative to negative control sequences. B, Candidate sequences can also be evaluated using genome-wide assays such as STARR-seq or MPRA (left). In these assays, fragmented genomic DNA is cloned (e.g. using Gateway Technology), into a reporter construct in the 3′-UTR (STARR-seq) or upstream of the gene (MPRA). With MPRA, barcodes are inserted into the open reading frame (pink vertical bar). The resulting reporter construct library is transiently transfected into protoplasts or infiltrated into *N. benthamiana* leaves. Activity of the candidate fragments (left) is evaluated by measuring reporter transcript abundance in comparison to the abundance of input plasmid (right). For MPRAs, next-generation sequencing of the plasmid library is used to pair the unique barcodes in the reporter gene with the inserted candidates.

Functional evaluation of the regulatory activity of putative CRMs poses a major challenge. The bottleneck is the low-throughput of transient and transgenic gain- or loss-of-function assays (van der Meer et al., 1992; Studer et al., 2011; Belele et al., 2013; Zhu et al., 2015; Du et al., 2020). In these experiments, putative CRMs are cloned up- or downstream of reporter genes. These reporters typically are driven solely by a minimal promoter, or in addition by an enhancer, to test activation and silencing of the reporter genes, respectively. Reporter gene activity is assessed by (1) transient transfection of protoplasts, (2) *Agrobacterium* infiltration into *Nicotiana benthamiana* leaves, or (3) stable insertion into a plant genome (Figure 4A;  Studer et al., 2011; Belele et al., 2013; Zhu et al., 2015; Zhao et al., 2018; Yan et al., 2019; Du et al., 2020; Jores et al., 2020; Lin et al., 2019). Loss-of-function approaches also include inhibiting CRM activity through RNA-directed DNA methylation or mutational approaches (Mette et al., 2000; Sidorenko et al., 2009; Rodríguez-Leal et al., 2017; Zicola et al., 2019; Wang et al., 2021). It is important to be aware that CRMs may be functionally redundant, masking their function when deleted (Osterwalder et al., 2018; Wang et al., 2021).

Clustered Regularly Interspaced Short Palindromic Repeats (CRISPR)-mediated genome editing is becoming a preferred tool to modify sequences in their endogenous genomic context; it can be used to introduce quantitative variation and reveal hidden pleiotropy in many crop traits (Rodríguez-Leal et al., 2017; Liu et al., 2021; Wang et al., 2021; Hendelman et al., 2021).

The high-throughput method self-transcribing active regulatory region-sequencing (STARR-seq), and variants of this approach, enable *en masse* evaluation of CRM activity of genomic fragments (Figure 4B;  Arnold et al., 2013; Inoue and Ahituv, 2015; Gisselbrecht et al., 2020; Pang and Snyder, 2020). In the STARR-seq assay, genomic fragments are cloned into the 3'-untranslated region (3'-UTR) of a reporter gene, creating a library of reporter constructs that is transiently transfected into cells. RNA-seq of reporter gene-derived transcripts indicates the fragments possessing enhancer activity as these will enhance their own transcription. STARR-seq has been adapted to rice and maize (Ricci et al., 2019; Sun et al., 2019). The study in maize validated that ACRs are enriched for enhancer activity, especially those possessing HAc at flanking nucleosomes (Ricci et al., 2019). A recent study (Jores et al., 2020) using *Agrobacterium* infiltration of reporter libraries into *N.* *benthamiana* leaves as well as maize protoplasts showed that enhancers were most effective when cloned upstream of the TSS, as in the massively parallel reporter assay (MPRA; Inoue and Ahituv, 2015), instead of in the 3′-UTR. Intriguingly, Jores et al. (2020) observed environmental responsiveness of three enhancers, showing that transient reporter assays can be used to test the responsiveness of CRMs to changing conditions. Although STARR-seq and MPRA both provide a highly scalable method for CRM assessment, the outcome is affected by (1) the minimal promoter used, (2) the evaluation of CRM activity in plasmids rather than in their native chromatin context (Inoue et al., 2017), (3) the cell types in the protoplast or leaf sample, and (4) the plant species used since not all CRMs are functional in other plant species (Belele et al., 2013).

### TF-assisted characterization of CRMs

Functional characterization of CRMs includes the identification of TFs binding to individual CREs within CRMs and the interplay between CRM-bound TFs. Methods applied can be divided into TF- and DNA-centered approaches (Yang et al., 2016; Springer et al., 2019). ChIP-seq is the gold standard to map genome-wide TF binding in vivo (Johnson et al., 2007; Kaufmann et al., 2009). Although there are publicly available TF ChIP-seq data in plants (Kaufmann et al., 2009; Morohashi et al., 2012; Chang et al., 2013; Eveland et al., 2014; Song et al., 2016; Alvarez et al., 2020; Tu et al., 2020), only a minor fraction of the TFs has been examined, and most data are from a single plant species, Arabidopsis. Reasons for so few data sets include (1) the limited availability of ChIP-grade antibodies, (2) the lack of transgenic plants expressing functional, epitope-tagged TFs, (3) the highly dynamic nature of TF–DNA interactions (Para et al., 2014), and (4) low-throughput of the method. One possibility is to transiently express epitope-tagged TFs to map genome-wide TF targets by ChIP-seq (Lee et al., 2017; Tu et al., 2020).

Besides in vivo approaches, there are high-throughput in vitro TF-centered assays, such as the use of protein binding microarrays (PBMs; Franco-Zorrilla et al., 2014; Mukherjee et al., 2004; Weirauch et al., 2014), and DNA-affinity purification-sequencing (DAP-seq; O’Malley et al., 2016; Bartlett et al., 2017). The drawback of both methods is the use of naked, rather than nucleosomal DNA. Consequently, TF binding specificity in vitro is not necessarily the same as that in vivo.

Besides TF-centered, there are also DNA sequence-centered approaches, of which yeast-one hybrid (Y1H) is most widely used (Meng and Wolfe, 2006; Gaudinier et al., 2011). This method identifies TFs (prey) interacting with a specific DNA sequence (bait) of interest. PBMs, DAP-seq, and Y1H require the use of full-length cDNA sequences for the TFs being assayed and are therefore limited to species with available TF cDNA clone collections (Burdo et al., 2014; Pruneda-Paz et al., 2014).

## Genomic location of CRMs: the influence of genome size and organization

CRMs are often computationally classified into a few major categories based on their linear distance to the nearest TSS of coding regions of genes. These categories generally include CRMs overlapping genic sequences (core promoter, 5′-UTR, exon, intron, 3′-UTR), and those within 2–5 kb of the TSS, 5-kb downstream of the transcription stop site, and further away from a gene (distal). Although a majority of the cis*-*CRMs are roughly within a few kbp upstream of their protein coding target genes (Wu et al., 2013; Shlyueva et al., 2014; Lu et al., 2019), numerous CRMs are located elsewhere, such as introns or more than 10 kbp away of their target genes, both upstream and downstream of genes (Oka et al., 2017; Wang et al., 2017; Li et al., 2019b; Lu et al., 2019; Ricci et al., 2019). For several species, including a number of plants, the average length of first introns is significantly larger than that of other introns (Bradnam and Korf, 2008), suggesting the presence of CRMs. Genome-wide experimental and computational studies in Arabidopsis showed enrichment for regulatory activity of first introns, although the genomic location seems more important than the intron size (Back and Walther, 2021; Meng et al., 2021). Larger intron size might result from selection favoring insertions over deletions as a way to preserve functional CREs. CRMs are, for example, found in large introns in *FLOWERING LOCUS C* and *AGAMOUS* in Arabidopsis and *knotted1* in maize (*Zea mays*; Greene et al., 1994; Sieburth and Meyerowitz, 1997; Busch et al., 1999; Hong et al., 2003; Qüesta et al., 2016; Yuan et al., 2016; Liu et al., 2020).

**Figure 5:**
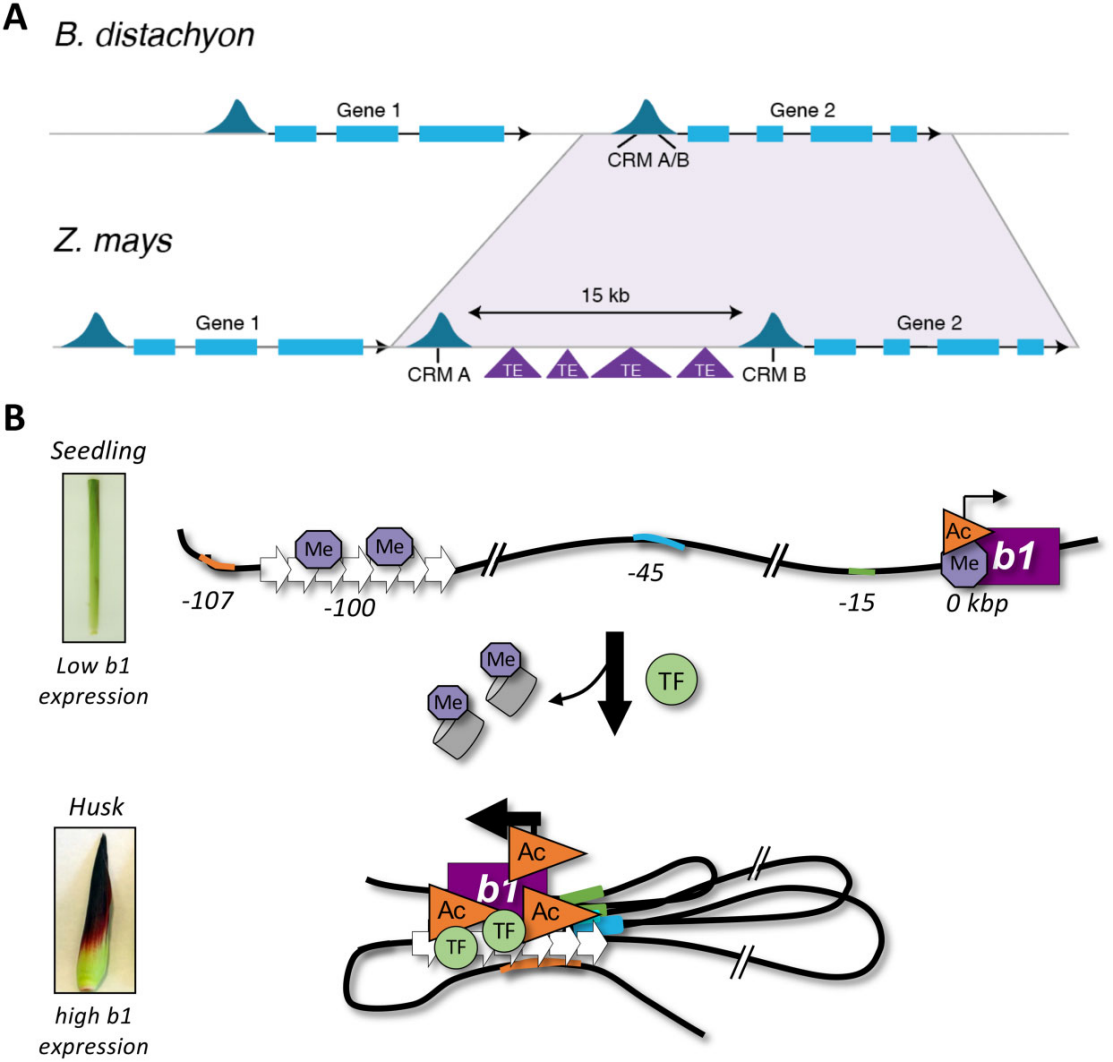
Large genomes: distant CRMs and chromatin interactions. A, The number and distance of CRMs to their target gene can increase in larger plant genomes. Hypothetical regions of accessible chromatin (dark blue peaks) are shown in a region of synteny between *B. distachyon* and *Z. mays*. The shaded light purple region indicates a gene with expanded intergenic space. The region of accessible chromatin for this gene in *B. distachyon* possesses one CRM (A/B) containing several CREs within a single accessible chromatin region, whereas in *Z. mays* the CREs within CRM (A/B) are split into two CRMs, A and B, through insertion of TEs. CRM A and B together carry similar CREs as CRM (A/B) in *B. distachyon*, but now separated by 15 kb of intergenic sequence. B, Chromatin interactions between distal CRMs and their target gene affect gene expression levels. An example of chromatin interactions that positively correlate with expression levels. The maize *Booster-Intense (B-I)* allele contains the *b1* hepta-repeat enhancer 100-kbp upstream of the *b1* TSS, and other putative CRMs at ∼15-, 45-, and 107-kbp upstream of the TSS. In seedling tissue of *B-I* plants, *b1* is lowly expressed, low H3K9ac and H3K27me2 levels are observed at the gene body, and low H3K27me2 levels at the enhancer. Upon transcriptional activation of *b1* in husk tissue, nucleosomes and H3K27me2 are lost at the enhancer and gene, H3K9ac levels increased, and the repeat enhancer and additional CRMs upstream physically interact with each other and the TSS of *b1*, resulting in enhanced *b1* expression. Orange triangles, histone acetylation; light purple octagonal shapes, H3K27me2; grey barrels, nucleosomes; green circles, TFs.

Classifications based on distance are somewhat arbitrary, but useful to conceptualize how CRM locations evolved within and between species. When using chromatin accessibility as a proxy for CRM activity, it becomes clear that differences in distance to the nearest gene are due to the variation in genome size. For example, in Arabidopsis, having a very small genome (∼135 Mbp), and rice, having a slightly bigger genome (∼373 Mbp), nearly 45% and ∼25%, respectively, of the ACRs are located within 1 kbp of their target gene (Zhang et al., 2012a, 2012b), while in maize (∼2,400 Mbp) most ACRs are located >1 kbp of the nearest gene (Rodgers Melnick et al., 2016). This relation between CRM location and genome size is confirmed by additional chromatin accessibility studies in angiosperms (Zhang et al., 2012a, 2012b; Oka et al., 2017; Wang et al., 2017; Maher et al., 2018; Zhao et al., 2018; Li et al., 2019b; Lu et al., 2019; Reynoso et al., 2019; Ricci et al., 2019). There are numerous explanations for the correlation between genome size and proportion of distal CRMs. The most likely explanation is that in larger genomes, CRMs that were adjacent to one another in smaller genomes, became separated in genome space by transposon proliferation and repeat expansion (Dong et al., 2018, 2017; Wang et al., 2018; Li et al., 2019a; Peng et al., 2019; Ricci et al., 2019). For example, in *Brachypodium distachyon* (∼355 Mbp), a cluster of CREs could be present in a single ACR within 500 bp of a target gene, yet in *Z.* *mays*, this same cluster might have split in two ACRs by one initial transposon insertion followed by more TE insertions over evolutionary time (Figure 5A). Regardless of the genome size, the number of genes and putative CRMs are highly correlated (Maher et al., 2018; Lu et al., 2019; Ricci et al., 2019). Large plant genomes do have more ACRs, and thus DNA sequence underlying ACRs. However, the number of ACRs does not vary more than about twofold between plant species ranging in genome size from ∼150 Mbp to ∼5,000 Mbp (Lu et al., 2019).

## Distal CRMs become functional through long-distance chromatin interactions

Distal CRMs—enhancers and/or silencers—affect the expression of their target genes through chromatin interactions (De Laat and Duboule, 2013; Louwers et al., 2009; Ricci et al., 2019; Doni Jayavelu et al., 2020; Gisselbrecht et al., 2020; Ngan et al., 2020; Pang and Snyder, 2020). ChIA-PET and Hi-ChIP experiments identified tens of thousands of H3K4me3-, H3K27ac-, or H3K27me3-centered long-range intra-chromosomal interactions between distal CRMs and core promoters in different maize and rice tissues (Li et al., 2019a; Peng et al., 2019; Ricci et al., 2019; Zhao et al., 2019a). A subset of these interactions is dynamic between different tissues, developmental stages, and environmental conditions (Louwers et al., 2009; De Laat and Duboule, 2013; Li et al., 2019a). For example, in maize, the interaction frequency between upstream CRMs and the *b1* gene positively correlates with the *b1* expression level (Figure 5B;  Louwers et al., 2009). Interestingly, it was noted that ∼40% of distal putative CRMs in maize did not interact with one of the immediate flanking genes, i.e. they tended to skip at least one gene and interact with more distal genes (Li et al., 2019a; Fagny et al., 2021). This is reminiscent of how DICE, a distal CRM, controls the expression of the maize *bx1* gene (Zheng et al., 2015). Mediator, a transcriptional coactivator complex, plays a key role in chromatin interactions between distal CRMs and core promoters in animals (Kagey et al., 2010). In Arabidopsis, jasmonic acid regulates chromatin interactions that are dependent on the mediator subunit MED25 (Wang et al., 2019), indicating a crucial role for Mediator in chromatin interactions in plants as well.

How do distal CRMs and target genes find each other in 3D space? Genome-wide studies using 3C technology have uncovered that chromatin regions displaying similar epigenomic landscapes have the tendency to physically interact with each other through a mechanism called phase-separation (Berry et al., 2017; Hnisz et al., 2017; Kim and Shendure, 2019; Stam et al., 2019; Zhao et al., 2019b; Zhang et al., 2020). Phase-separation is mediated by proteins such as HP1α and its plant homologue Agenet Domain Containing Protein 1 (ADCP1), but also RNAPII and Like Heterochromatin Protein 1 (Kim et al., 2021). As a consequence, active chromatin (active genes), facultative heterochromatin (PcG-silenced genes), and classical heterochromatin (silenced TEs) are organized into their own spatially separated territories, called topologically associated domains (TADs; Doğan and Liu, 2018; Rada‐Iglesias et al., 2018; Rowley and Corces, 2018; Stam et al., 2019). TADs are based on chromatin conformation data, and are computationally defined genomic sequences that have greater contact frequencies with one another than with sequences in neighboring domains. DNA sequences within one and the same TAD display higher interaction frequencies with each other than with sequences in other TADs. In animals, CRM–gene interactions appear mostly confined to TADs, with the borders between different TADs functioning as insulators (Rada‐Iglesias et al., 2018; Rowley and Corces, 2018). In plants, however, there are no indications for genuine insulator elements (Heger and Wiehe, 2014) and long-range chromatin interactions spanning TADs have been observed accordingly (Dong et al., 2017).

Numerous chromatin interactions have been identified by 3C-based experiments (Ricci et al., 2019; Dong et al., 2017; Li et al., 2019a; Peng et al., 2019). For example, P–P interactions appear frequent, and genes involved in such interactions tend to be enriched for co-expression (Li et al., 2012, 2019a; Peng et al., 2019; Zhao et al., 2019a). The observed P–P interactions may be due to gene co-regulation, but may also be due to phase separation, i.e. clustering of DNA sequences that have a similar chromatin state but do not affect each other’s expression (Hnisz et al., 2017; Kim and Shendure, 2019; Stam et al., 2019). Part of the P–P interactions may also be explained by part of the core promoters showing enhancer activity, regulating distal genes (Dao et al., 2017; Diao et al., 2017; Mikhaylichenko et al., 2018; Andersson and Sandelin, 2020). Distinguishing interactions that regulate gene expression from chromatin interactions due to physical proximity is required to allow defining distal CRM–gene pairs unequivocally.

## TEs: a major source of CRMs

### TE-derived CRMs

TEs can influence gene expression through several different mechanisms, including the disruption of CRMs, spreading of silent chromatin into flanking genes, and by providing novel CRMs (Hirsch and Springer, 2017). Here, we focus on the latter events. It is increasingly recognized that TEs provide a source of CRMs (Lisch, 2013; Chuong et al., 2017). It is estimated that about 25% and 30% of regulatory sequences could be TE-derived (TE-CRMs) in humans and maize, respectively (Oka et al., 2017; Zhao et al., 2018; Pehrsson et al., 2019; Fagny et al., 2021). Accordingly, several TEs are bound by TFs in mammals and plants (Bourque et al., 2008; Kunarso et al., 2010; Lynch et al., 2011; Batista et al., 2019). Transient reporter assays in mammalian and maize cells showed that a significant number of the TE-CRMs tested indeed showed enhancer or silencer activity (Lynch et al., 2011; Xie et al., 2013; Zhao et al., 2018). For maize, 8 out of 10 TE-ACRs tested drove reporter gene expression (Zhao et al., 2018). Thus, although most TEs appear silenced in most tissues, a significant fraction may have co-opted a regulatory function (Chuong et al., 2017; Pehrsson et al., 2019).

Specific to TE-CRMs is their potential to move and amplify within a genome. Accordingly, species-specific putative CRMs are often enriched in TEs (Lu et al., 2019). New insertions with favorable effects can be evolutionarily conserved, allowing rewiring of gene regulatory networks and the establishment of new gene networks during evolution, while insertions with unfavorable effects are likely to be neutralized by mutations (Hénaff et al., 2014). Rewiring of gene networks has been demonstrated for example for human embryonic stem cells, and networks involved in mammalian pregnancy and seed development in Arabidopsis (Feschotte, 2008; Kunarso et al., 2010; Chuong et al., 2017; Lynch et al., 2011; Batista et al., 2019). In general, proportionally older TEs seem to provide regulatory activity more often than younger TEs (Simonti et al., 2017; Pehrsson et al., 2019). The notion is that the older the TEs, the more time there was for CRMs to lose DNA methylation and co-opt a regulatory function for the host. Remarkably, certain TE families, such as endogenous human and mouse retroviruses, show greater regulatory potential than others (Simonti et al., 2017; Pehrsson et al., 2019). In plants, in absolute numbers, more putative CRMs are located in retroviruses than in DNA transposons (Oka et al., 2017; Zhao et al., 2018, 2020; Lu et al., 2019). However, when analyzing the enrichment within these two classes in a range of plant species, putative CRMs are more enriched than expected in DNA transposons, and especially the hAT subclass (Zhao et al., 2018; Lu et al., 2019). It is important to note that the number of TE-CRMs may be significantly underestimated. Most genome-wide analyses use unique mapping of short reads to a reference genome, which prevents read mapping to repetitive TE sequences (Panda and Slotkin, 2020). Sequencing of longer paired-end reads increases the ability to map sequence reads unambiguously (Panda and Slotkin, 2020). In addition, as is true for other CRMs, TEs acting as tissue-, cell type-, or stress-specific CRMs will only be detected when analyzing the relevant tissues, cell types, and conditions.

### Examples of TE-related CRMs

**Figure 6:**
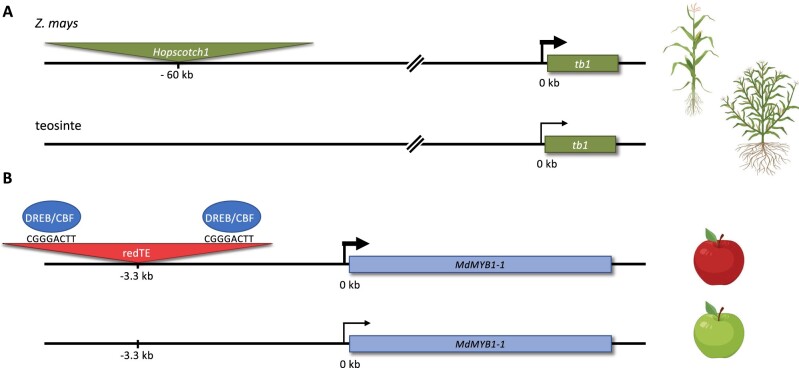
Examples of transposable elements acting as regulatory sequences. A, In *Z. mays*, a *Hopscotch* retrotransposon inserted ∼60-kbp upstream of *tb1.* This TE is absent in teosinte (Studer et al., 2011). The *Hopscotch* TE acts as an enhancer of *tb1* expression and partially explains the increased apical dominance observed in *Z. mays* versus teosinte. B, In apple, a 4-kbp Gypsy-like retrotransposon, redTE, inserted upstream of the *MdMYB1-1* gene, increases the expression of this gene, resulting in red-skinned apples (Zhang et al., 2019). RedTE contains the “GCCGACTT” CRE, a TFBS for a DREB/CBF TF that enhances the expression level of *MdMYB1* at low ambient temperatures. Created using Biorender.com.

Although genome-wide assays suggest abundant TE-CRMs in multiple plant genomes (Makarevitch et al., 2015; Oka et al., 2017; Zhao et al., 2018; Lu et al., 2019; Zhao et al., 2020a), there are relatively few well-described examples in which a specific phenotype is connected with TE-CRMs (see e.g. Lisch, 2013; Hirsch and Springer, 2017). A classic example is the *Hopscotch* retrotransposon enhancing the expression levels of its target gene *tb1* in domesticated maize compared to its wild relative teosinte (Figure 6A;  Studer et al., 2011). Using STARR-seq, enhancer activity was detected from part of the *Hopscotch* element (Ricci et al., 2019). In Arabidopsis, deletion of one out of four TEs in the promoter of a Jacalin Lectin family protein gene changed root-specific expression into a constitutive expression pattern (Wu et al., 2018), indicating a silencer function for the deleted TE. An example of a TE disrupting CRM function, is a *MITE* TE insertion in *Vgt1*, a putative enhancer of the maize floral repressor gene *ZmRap2.7* (Salvi et al., 2007). This *MITE* insertion is associated with early flowering.

For a number of TE-CRMs, the TFBSs involved in regulating associated target genes are known (Yokosho et al., 2016; Barco et al., 2019; Zhang et al., 2019). For example, in *Malus domestica* (apple), a 4-kbp retrotransposon, *redTE*, increases the expression level of *MdMYB1*, resulting in red skin color of the fruits (Figure 6B;  Zhang et al., 2019). *RedTE* contains a binding motif for a dehydration-responsive element/C-repeat-binding (DREB/CBF) TF that enhances *MdMYB1* expression at a relatively low ambient temperature. Strikingly, in maize, TEs associated with cold-, heat-, and salt-induced expression of nearby genes are also shown to be enriched for DREB/CBF TFBSs (Makarevitch et al., 2015). Similarly, the ONSEN transposon in Arabidopsis harbours heat-responsive CREs that recruit HEAT SHOCK TRANSCRIPTION FACTOR A2 for activation of nearby genes (Ito et al., 2011; Cavrak et al., 2014).

Although DNA methylation patterns appear very stable in plant tissues (Schmitz et al., 2013; Li et al., 2015; Kawakatsu et al., 2016; Oka et al., 2020; Crisp et al., 2020), there are indications that DNA demethylation of TE-CRMs may enhance the expression of particular stress-responsive genes upon application of various types of stresses (Dowen et al., 2012; Yua et al., 2013). For example, the expression of *RESISTANCE METHYLATED GENE 1* by the bacterial flagellin-derived peptide flg22 is associated with DNA demethylation of two *helitron*-related TE repeats upstream of the coding region by the DNA demethylase REPRESSOR OF SILENCING 1. These results suggest that DNA demethylation of TE-CRMs may play a role in plant immune responses. It remains unclear if the observed changes in DNA methylation at flanking TEs are causally related to the upregulation of linked stress-responsive genes. It has been shown that changes in gene expression can lead to changes in DNA methylation levels of nearby TEs (Secco et al., 2015).

## Sequence conservation of CRMs in plants

**Figure 7:**
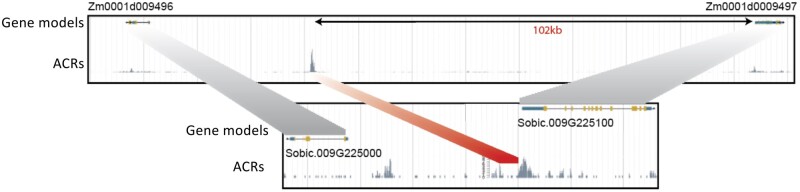
Conserved non-coding sequences underlying ACRs show distinct locations relative to target genes between species. An example of a syntenic region between maize and sorghum. The region depicts two orthologous pairs of genes (linked by gray shading). An ACR detected in both species contains conserved DNA sequences (linked by red shading). Although the ACR in sorghum is near the TSS of Sobic.009G225100, the homologous ACR in maize is located more than 102 kb away from the orthologous gene, Zm001d0009497.

Sequence diversification of CRMs is a crucial factor underlying phenotypic variation between and within species (Long et al., 2016). Functional diversification of CRMs, but also genes, and thereby gene regulatory networks, is driven by species hybridization and whole-genome duplications (Ramírez-González et al., 2018; Jones and Vandepoele, 2020). Focusing on CRMs, one of the copies can be lost, or both copies diversify, leading to neo- and subfunctionalization (Arsovski et al., 2015; Long et al., 2016). Indeed, studying the fate of duplicated CRMs over time has indicated that retention of CRMs between paralogs negatively correlates with divergence time from the duplication event. Analysis of paralogous distal ACRs in *Z.* *mays* and *Glycine max* revealed that, for ∼50% of them (>50% in *G. max)*, both were accessible in the tissues studied (Lu et al., 2019). In species pairs such as *Z.* *mays*–*Sorghum bicolor* and *G.* *max*-*Phaseolus vulgaris*, over half of the distal CRMs are shared within the pairs, and two thirds of these possess accessible chromatin in both species (see an example in Figure 7;  Lu et al., 2019). The diversification of duplicated CRMs is reflective of various factors, including domestication, selection, the timing of genome duplications, and the rates of sequence loss and changes (Freeling et al., 2015; Cheng et al., 2018).

The rate of sequence diversification at CRMs depends on their function. A number of CRMs have been conserved during evolution, indicating they represent functionally important CRMs; comparative analyses can be used to identify such CRMs. Long divergence times enable sequence turnover, especially in non-coding regions, revealing CRMs containing CNS that have been preserved through time. In agreement, CNS are enriched in accessible chromatin regions and putative enhancers (Zhang et al., 2012a; Oka et al., 2017; Lu et al., 2019; Hendelman et al., 2021).

Early efforts to identify CRMs using comparative approaches relied on locus-specific phylogenetic shadowing, whereby closely related species are used to find additive genetic differences to identify significant regions of conservation (Wittkopp and Kalay, 2012). This approach led to the identification of multiple CRMs in plants, for example at the *AG* and *FLOWERING LOCUS T (FT)* loci in Arabidopsis, and *MIR164* locus in *Brassica* (Hong et al., 2003; Adrian et al., 2010; Jain et al., 2018). The intron of the *AG* gene even contains multiple conserved TF motifs among several Brassicaceae. Phylogenetic shadowing is also being applied genome-wide, finding CNS by comparing syntenic orthologous sequences (Maher et al., 2018; Lu et al., 2019; Reynoso et al., 2019). Synteny is often key to identify CNS genome-wide (Kaplinsky et al., 2002; Liang et al., 2018).

Although CNS are useful to identify conserved versus diversified CRMs, the rate of nucleotide substitutions can limit the detection of CRMs. Therefore, many CRMs will require other data, such as chromatin structure data, to pinpoint their location (Shlyueva et al., 2014; Weber et al., 2016; Lu et al., 2019). Altogether, information on sequence conservation of CRMs supports the idea that non-coding sequence variation contributes to phenotypic variation with evolutionary consequences for plant diversification and adaptation.

## Future challenges and perspectives

This is an exciting time to study cis-regulatory sequences in plant genomes. However, there are still many challenges to be overcome to improve the discovery, characterization, and functional evaluation of CRMs, and the identification of their target genes.

A major challenge is the discovery of tissue- and condition-specific plant CRMs. This likely will be met by emerging single-cell droplet-based assays (Buenrostro et al., 2015; Dorrity et al., 2021; Farmer et al., 2021). Single-cell RNA-seq has already been implemented in plants by several groups (Efroni et al., 2016; Libault et al., 2017; Denyer et al., 2019; Jean-Baptiste et al., 2019; Shulse et al., 2019; Ryu et al., 2019), and single-cell ATAC-seq studies in Arabidopsis and maize have been reported (Dorrity et al., 2021; Farmer et al., 2021; Marand et al., 2021). Whereas single-cell ChIP-seq, Hi-C, and whole-genome bisulfite sequencing methods are emerging for mammalian systems (Ramani et al., 2017; Luo et al., 2018; GrosseLin et al., 2019; Lee et al., 2019; Zhou et al., 2019), they have yet to be implemented for plants. The combination of different single-cell data sets will be especially powerful in revealing the cis-regulome of plant genomes.

Another challenge will be to identify which putative CRMs are enhancers versus silencers and which combine both functions. It is important to fill this knowledge gap as relatively few silencers have been identified and confirmed in plants to date. In addition, a minimum set of marks and/or proteins should be identified that distinguish enhancers from silencers, and ideally also their different activity states. One should not underestimate the efforts required to accomplish this.

A third challenge is the detection of CRM–gene pairs, especially important since significant numbers of CRMs may regulate genes other than the immediate flanking genes. The use of single-cell 3C-based technology for this purpose would be extremely challenging. Fortunately, single-cell ATAC-seq in combination with single-cell RNA-seq can be used to exploit co-accessibility of ACRs and expression levels of putative target genes across cell types (Pliner et al., 2018; Ma et al., 2020). Recent data from such an approach showed that predicted CRM–gene pairs in maize leaf cell-types coincided with about 78% of the Hi–C interactions detected in maize leaf tissue (Marand et al., 2021). Predicting CRM–gene pairs is further complicated by the opposite correlations expected for enhancers and silencers.

Identifying the TFs and cofactors bound and tethered to CRMs also remains a significant challenge, because the gold standard approach, ChIP-seq, is time consuming and difficult to implement in systems with limited transformation efficiency. Even a plant with a small genome like Arabidopsis could potentially have 5–20 million TF–CRM interactions to validate (Ouma et al., 2018).

Plant researchers are going through a discovery phase concerning the cis-regulomes of different plant species, but in the future our attention will turn to applying this knowledge. Including CRMs in genome editing pipelines will complement existing efforts to edit gene-coding sequences and improve plant trait performance. Depending on the continent and plant species used, endogenous CRMs may be engineered through CRISPR-based mutagenesis, conventional mutagenesis methods, or crossing in existing genetic variants. In addition, CRMs, including synthetic CRMs, will be used to express transgenes encoding proteins of interest in a cell-type-specific manner, or to rewire existing TF networks. In the meanwhile, natural and induced genetic variation in CRMs will continue to be linked to existing and novel phenotypic variation.

## Notes

All three authors contributed to writing and approved the final version. M.S. integrated all sections of the text. R.J.S. and M.S. created the figures.

The author(s) responsible for distribution of materials integral to the findings presented in this article in accordance with the policy described in the Instructions for Authors (https://academic.oup.com/plcell) is: Maike Stam (m.e.stam@uva.nl)
